# Birth-Preparedness for Maternal Health: Findings from Koupéla District, Burkina Faso

**Published:** 2006-12

**Authors:** Allisyn C. Moran, Gabriel Sangli, Rebecca Dineen, Barbara Rawlins, Mathias Yaméogo, Banza Baya

**Affiliations:** ^1^ Department of International Health, Johns Hopkins Bloomberg School of Public Health, Baltimore, MD, USA and ICDDR,B, GPO Box 128, Dhaka 1000, Bangladesh; ^2^ Institut Supérieur des Sciences de la Population, Ouagadougou, Burkina Faso; ^3^ JHPIEGO, Baltimore, MD; ^4^ BASICS, Rwanda

**Keywords:** Maternal health, Birth-preparedness, Delivery, Childbirth, Burkina Faso, West Africa

## Abstract

Maternal mortality is a global burden, with more than 500,000 women dying each year due to pregnancy and childbirth-related complications. Birth-preparedness and complication readiness is a comprehensive strategy to improve the use of skilled providers at birth, the key intervention to decrease maternal mortality. Birth-preparedness and complication readiness include many elements, including: (a) knowledge of danger signs; (b) plan for where to give birth; (c) plan for a birth attendant; (d) plan for transportation; and (e) plan for saving money. The 2003 Burkina Faso Demographic and Health Survey indicated that only 38.5% of women gave birth with the assistance of a skilled provider. The Maternal and Neonatal Health Program of JHPIEGO implemented a district-based model service-delivery system in Koupéla, Burkina Faso, during 2001–2004, to increase the use of skilled providers during pregnancy and childbirth. In 2004, a cross-sectional survey with a random sample of respondents was conducted to measure the impact of birth-preparedness and complication readiness on the use of skilled providers at birth. Of the 180 women who had given birth within 12 months of the survey, 46.1% had a plan for transportation, and 83.3% had a plan to save money. Women with these plans were more likely to give birth with the assistance of a skilled provider (p=0.07 and p=0.03 respectively). Controlling for education, parity, average distance to health facility, and the number of antenatal care visits, planning to save money was associated with giving birth with the assistance of a skilled provider (p=0.05). Qualitative interviews with women who had given birth within 12 months of the survey (n=30) support these findings. Most women saved money for delivery, but had less concrete plans for transportation. These findings highlight how birth-preparedness and complication readiness may be useful in increasing the use of skilled providers at birth, especially for women with a plan for saving money during pregnancy.

## INTRODUCTION

Maternal mortality is a substantial burden in developing countries. The World Health Organization (WHO) estimates that 500,000–600,000 women die from pregnancy and childbirth-related complications each year, with 99% of these deaths occurring in developing countries (1). In sub-Saharan Africa, the maternal mortality ratio is 920 per 100,000 livebirths, and the lifetime risk of maternal death is 1 in 16 compared to 1 in 2,400 in Europe (1). Improving maternal mortality has received recognition at the global level as evidenced by the inclusion of reducing maternal mortality in the Millennium Development Goals (2).

Since it is not possible to predict which women will experience life-threatening obstetric complications that lead to maternal mortality, receiving care from a skilled provider (doctor, nurse, or midwife) during childbirth has been identified as the single most important intervention in safe motherhood (3). However, the use of skilled providers in developing countries remains low. According to the demographic and health surveys, only 51% of women in developing countries were assisted by a skilled provider at last birth (4).

Thaddeus and Maine outlined three delays that influence the provision and use of obstetric services to prevent maternal deaths: (a) delay in deciding to seek care if complication occurs; (b) delay in reaching care; and (c) delay in receiving care (5). The Maternal and Neonatal Health (MNH) Program of JHPIEGO developed the birth-preparedness and complication readiness matrix to address these three delays at various levels, including the pregnant woman, her family, her community, health providers, health facilities, and policy-makers during pregnancy, childbirth, and the postpartum period. The concept of birth-preparedness and complication readiness includes knowing danger signs, planning for a birth attendant and birth-location, arranging transportation, identifying a blood donor, and saving money in case of an obstetric complication (6). Birth-preparedness and complication readiness is a key strategy in safe motherhood programmes; however, there is no evidence of its effectiveness in improving maternal morbidity or mortality (7). This paper explored the elements of birth-preparedness and complication readiness and their association with increased use of a skilled provider at birth in one district in Burkina Faso.

## MATERIALS AND METHODS

### Study site

Burkina Faso has one of the highest maternal mortality ratios in the world at 1,000 maternal deaths per 100,000 livebirths (1), and only 38.5% of women give birth with the assistance of a skilled provider (8). To address the high levels of maternal mortality, in 1998, the Ministry of Health initiated a national safe motherhood programme. The MNH Program supported this national effort by implementing a comprehensive district-based programme in Koupéla in collaboration with Plan, United Nations Children's Fund (UNICEF), and a consultant from Mwangaza Action, a local non-governmental organization. Koupéla was selected for the MNH Program because of the opportunity for maxi-mizing limited resources through collaboration with Plan and UNICEF—two key international organizations that were already working in safe motherhood and child survival in the district. The Program worked in 13 of the 24 health facilities and their surrounding communities.

The activities of the MNH Program were designed to improve the quality and use of health services. Activities were identified through a hands-on problem-solving approach called “performance and quality improvement (PQI).” The approach involved working with all stakeholders in maternal health (pregnant women, community members, health providers, and policy-makers) to identify and analyze gaps in maternal health services and offer locally-adapted solutions to reduce these gaps. Through this process, the stakeholders identified a number of key activities, including training health workers, providing essential equipment and supplies, and strengthening supervision by the district health-management team. The process also underlined the need to strengthen the linkages between health centres and the community, such as improving communication systems among doctors, midwives, community members, and traditional birth attendants, developing emergency transportation, and financing plans for health centres. Regular meetings between traditional birth attendants and facility-based health providers were held to foster trust and better working relationships.

Using PQI at the community level, the community members identified gaps in safe motherhood and developed community-based action plans. Action plans included organizing events around Mother's Day and Malaria Action Day to improve awareness of maternal and newborn health. Involvement of the community members in PQI also ensured that healthcare providers, community-based health workers, and traditional birth attendants provided one-on-one counselling with pregnant women and their families on key messages focused on birth-preparedness and complication readiness and recognition of danger signs using a flipchart. These messages were reinforced through district-based radio messages.

The MNH Program was evaluated using a pre-post-no control study design. A baseline survey was conducted during July-September 2001, and a follow-up survey was completed during February-March 2004 to measure exposure to the MNH Program and programme effectiveness. An independent research organization—Institut Supérieur des Sciences de la Population (ISSP)—conducted the baseline and follow-up surveys with technical input from the MNH Program.

### Sample

The study area comprised 145 villages in 13 health facility catchment areas. Within each catchment area, villages were stratified as further than the average distance to the referral health centre or within the average distance to the referral health centre. Three villages within each stratum were randomly selected, with five villages randomly selected from Koupéla and Pouytenga catchment areas to ensure adequate representation. Within these selected villages, pregnant women (n=180) and women who gave birth within 12 months of the survey (n=180) were randomly selected. Women were asked about knowledge, attitudes, and practices with regard to maternal and newborn health, including sociodemographic information and exposure to the MNH Program interventions. In-depth interviews with 30 women who had given birth in the 12 months prior to the survey were conducted to better understand the elements of birth-preparedness and complication readiness. In this paper, qualitative and quantitative data from the follow-up survey are presented.

The quantitative survey took about one hour to administer, and the qualitative interview took approximately 45 minutes to complete. This study was reviewed and approved by Western Institutional Review Board in the United States and the Burkina Faso Ministry of Health to ensure the protection of human subjects. All women in the study gave verbal consent prior to enrollment.

### Measures

Research demonstrates that higher wealth, urban residence, and maternal education are strongly associated with giving birth with the assistance of a skilled provider in a facility (9, 10). Use of antenatal care and use of health facilities for previous births are strongly associated with use of services (11). Proxy measures for wealth included household assets, such as household ownership of a radio, mode of household transportation, and principal employment for the head of the household.

#### Exposure

Exposure to the MNH Program interventions was measured by asking women if they had heard the term ‘birth-preparedness’, and if yes, the source of the exposure. Women who heard the term from an MNH Program channel were categorized as exposed to the intervention. The MNH Program channels included healthcare providers, community mobilization agents of MNH, from MNH flipcharts, from MNH village theatre, from White Ribbon events, or through district-based radio messages of MNH.

#### Birth-preparedness and complication readiness

Birth-preparedness and complication readiness was measured using a series of questions about knowledge of danger signs; plans for a birth provider; plans for transport in case of emergency; and plans for saving money in case of emergency.

**Knowledge of danger signs:** Women were asked to spontaneously cite danger signs during pregnancy, childbirth, the immediate postpartum, and for the newborn (four questions). An index of knowledge of danger signs was calculated as the number of pertinent danger signs spontaneously recalled by participants for each of these four periods (range=0 to 18). Since this response was spontaneous, only ‘correct’ responses were included in the index. These correct responses were chosen by a group of nurses, midwives, and physicians at the MNH Program. For pregnancy, responses included: bleeding, severe headache, trouble with vision, fever, swollen hands/face, and reduced or accelerated foetal movement. For childbirth, danger signs included: abdominal pain, prolonged labour, premature rupture of membranes, severe bleeding right after birth, and trouble with vision. For postpartum, danger signs included: severe bleeding, loss of consciousness, swollen hands, trouble with vision, and fever. For the newborn, danger signs included: difficulty breathing, blue or yellow appearance, difficulty breastfeeding, fever, and blood on the umbilical cord. The reliability of this index is within acceptable limits (Cronbach's alpha=0.81) (12).

**Plan for a birth attendant:** Women were asked about the birth attendant they planned to use during delivery. Doctor, nurse, midwife, or auxiliary nurse were coded as skilled birth attendants, while all others, including trained traditional birth attendants, were coded as non-skilled birth attendants.

**Plan for transportation:** All women were asked about plans for transportation in the case of emergency during their delivery.

**Plan for saving money:** All women were asked about plans for saving money in the case of emergency during their delivery.

### Data analysis

Quantitative data were entered into Integrated System for Survey Analysis (ISSA) and analyzed using SPSS (version 12.0) (SPSS, Chicago, Illinois) and Stata (version 8.0) (Stata Corporation, College Station, Texas). All qualitative interviews were translated into French from audio recordings and entered in English into MicroSoft Word. The NVIVO software was used for coding and organizing data around the birth-preparedness and complication readiness themes. Based on the findings from in-depth interviews, a model was developed for testing if birth-preparedness actions were associated with using a skilled provider at birth among recently-delivered women.

Unadjusted odds ratios were calculated to test for significant bivariate associations. Multiple logistic regression was used for examining significant associations between birth-preparedness and complication readiness variables and use of a skilled provider, controlling for confounders.

## RESULTS

### Background characteristics

The background characteristics of the pregnant and recently-delivered women in the quantitative survey and qualitative interviews were very similar, with the exception of parity and number of antenatal care visits. In the sample of pregnant women, there were more primiparous women with fewer antenatal care visits. Women were at different stages of pregnancy during the survey and, thus, may not have had the opportunity to complete antenatal care visits (Table 1).

**Table 1.** Percent distribution of study women by background characteristics and birth-planning behaviours, Koupéla, Burkina Faso, 2004

Characteristics	Quantitative survey				Qualitative interviews	
	Recently-delivered women (n=180)		Pregnant women (n=180)		Recently-delivered women (n=30)	
	No.	%	No.	%	No.	%
Age (years) (mean, SD)	27.8 (6.7)		27.8 (6.7)		29.2 (7.3)	
Education level						
None	164	91.1	150	83.3	26	86.7
Primary	12	6.7	28	15.6	5	13.3
Secondary+	4	2.2	2	1.1	0	0.0
Religion						
Islam	93	52.0	79	43.9	15	50.0
Protestant or Catholic	87	48.0	101	56.1	15	50.0
Ethnicity						
Mossi	160	88.9	156	86.7	22	73.3
Other	20	11.0	24	13.3	8	26.7
Marital status						
Monogamous	121	67.2	121	67.2	18	60.0
Polygamous	59	32.8	59	32.8	12	40.0
Parity						
0–1	35	19.4	60	33.3		
2–3	60	33.3	52	28.9		
4–5	37	20.6	35	19.4		
6 or more	48	26.7	33	18.3		
Principal employment						
Agriculture	148	82.2	145	80.6		
Other	32	17.8	35	19.4		
Transportation in household						
None	21	11.7	7	3.9		
Bicycle	125	69.4	133	73.9		
Motorized	34	18.9	40	22.2		
Women with radio in household	148	82.2	137	76.1		
No. of antenatal care visits (mean, SD)	3.14 (1.16)		1.61 (1.21)			
Distance to health centre						
Near	90	50.0	92	51.1		
Far	90	50.0	88	48.9		
Total	180	100	180	100	30	100

### Qualitative interviews

The results of in-depth interviews revealed that women understood the components of birth-preparedness and complication readiness and mentioned antenatal care services as a means for ensuring good health for their babies. They heard of the concept from auxiliary midwives during antenatal care visits and from radio transmissions.

Most women planned to give birth in a health centre with a skilled provider to ensure ‘health security’. Women stated that complications can arise at home, and health workers in the health centres are more qualified and competent to handle and refer these complications.

A 23-year old woman described the benefits of giving birth in a health centre as follows: “I did not choose to give birth at home because there are a number of risks and dangers. On the other hand, these risks are less at the health centre. In addition, at the CSPS [health centre], there is a possibility of referral to a higher level…giving birth at home with the assistance of older women can cause death of the baby or even the mother during the birth.”

Another woman, aged 24 years, stressed her opinion about the danger of birth at home: “A woman who gives birth at home can easily loose her life because of that. Sometimes you give birth at home and you find that there is a problem of blood, and they do not know what to do, so the person dies. At the CSPS [health centre], if after the birth you have a problem of blood, the agents [health workers] know…although at home there are problems that you are going to meet. That is why when I went to give birth, my husband said to go to the CSPS.”

Although women had positive attitudes towards giving birth in health centres, they often encountered barriers reaching facilities. Distances from villages to health centres were often long; women travelled 22.8 km, on average, through remote, rural areas to reach a health facility.

One woman stated: “I gave birth at home because the health centre is far away and it was during the rainy season. During the rainy season, two bodies of water enclose us and since it was during the night, if I went to the health centre, I would not be able to arrive there…even if you are with a moped, during the rainy season you cannot arrive. The person risks giving birth on the way and, there, that would be me on the way.”

Women were sometimes frustrated with their inability to follow through with their plans to give birth in a health facility. One 24-year old woman stated: “My husband and I prepared all. We bought a layette for the baby, a bucket, and other materials. When I sensed the birth, I went to the CSPS, and there, they told me that it was not labour but malaria accompanied by diarrhoea. They treated me and told me that they could not keep me because the labour had not started. The CSPS was burglarized, so they did not want to keep me, and they gave me an injection and asked me to go back home. The same evening I sensed the same pain and I thought that it was malaria that was getting worse. In the middle of the night, the pain got even worse, and then I understood that it was not malaria but it was the birth. So, in this way, I gave birth at home against my wishes.”

Although many women planned to give birth in a health centre, few women made plans for transportation. Women assumed that transportation would be available either from a family member or from a neighbour when needed and, therefore, did not feel the necessity of planning for transportation in advance.

A 35-year old explained: “I do not have any mode of transport. But there are certain people in the family who will help bring you in case you need. I thought that, at the time, I would feel the labour starting, within the family, I could find someone who would take me to the health centre. Since we live together, there are those who have a method of transportation and she goes with them.”

Women consistently mentioned saving money during their pregnancy for antenatal care, unforeseen costs due to birth-related complications, buying essentials, such as foods, soap, and clothes for the mother and newborn, and a means to avoid borrowing money from others. Women generated income for their savings plans mostly through agricultural and market activities, including selling millet, peanuts, *dolo* (local millet beer), and shea butter. Their husbands who saved money also had savings plans through agriculture and raising livestock. Several women mentioned learning about the importance of financial planning and how to save money at antenatal care visits. Healthcare workers explained a method that almost every woman revealed—save half of what is earned for the day and spend the other half.

A 39-year-old asserted the importance of a savings plan: “It is good, something that I thought about and planned. I say that when I knew I was pregnant that it was necessary to save five francs every time I earned 15 francs. This initiative was to help me face the needs of the expenses that would come with the pregnancy, during the birth, and even after the birth. In saving the money, this helped me to have financial means to do what necessitates in any the case of need, that is what I did.”

### Quantitative survey

These themes of planning for a birth assistant, planning for transportation, and saving money in the case of an emergency were further explored in the quantitative survey. Between the baseline and the follow-up survey, the use of a skilled provider at birth increased from 38.9% to 57.8% in the study area, and we were interested in understanding the relationship between birth-preparedness actions and use of skilled providers at follow-up.

Knowledge of danger signs was not commonly mentioned in the qualitative interviews—however, these messages were a key part of the programme and were explored in the quantitative survey. Among recently-delivered women, knowledge of danger signs was relatively low, with women spontaneously reporting an average of 5.67 danger signs in pregnancy, childbirth, after delivery, and for the newborn. The majority of women reported planning for birth; 43.4% planned for a birth provider, 46.1% planned for transportation, and 83.3% planned to save money in the case of an emergency. Birth-planning was also high among pregnant women. Seventy-one percent planned for a birth provider, 51.1% planned for transportation, and 61.1% planned to save money in the event of an emergency (Fig. 1). Planning for a provider and saving money were significantly different among pregnant women and recently-delivered women (p=0.000; p=0.000 respectively). Although women in later stages (6–9 months) of pregnancy were more likely to have initiated planning activities than women in the first five months of pregnancy, the differences were not statistically significant (data not shown).

**Fig.**
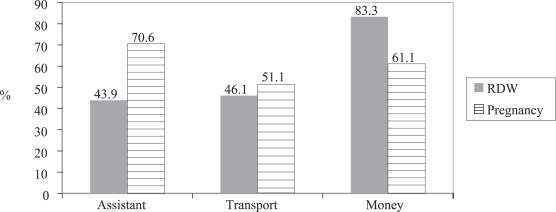
Birth-preparedness behaviours of recently-delivered women (n=180) and currently-pregnant women (n=180), Koupéla district, Burkina Faso, 2004

RDW=Recently-delivered women

In bivariate analysis, household assets, principal source of employment, religion, ethnicity, marital status, parity, and education of women were not significantly associated with using a skilled provider at delivery. Of birth-preparedness behaviours, only saving money for emergencies was significantly associated with giving birth with the assistance of a skilled provider (p=0.03). Women living further from the health centre were less likely to use a skilled provider at delivery (p=0.007), and women with more antenatal care visits were more likely to use a skilled provider (p=0.003).

In multivariate analysis, use of antenatal care and distance from the health facility were included in the final model since they were important themes in qualitative interviews and highly significant at the bivariate level. We also included education of women and parity as the literature demonstrates that these variables are strongly associated with use of skilled care at delivery.

Each of the birth-planning variables was added to the model one at a time. Women with knowledge of at least five danger signs were no more likely to give birth with the assistance of a skilled provider than women with less knowledge of danger signs (odds ratio [OR]=1.08; 95% confidence interval [CI] 0.55–2.01). Similarly, women with a plan for transport were no more likely to give birth with the assistance of a skilled provider than those women who did not plan for transport (OR=1.71; 95% CI 0.78–3.76). Interestingly, women who planned to give birth with the assistance of a skilled provider were 53% less likely to actually use a skilled provider at delivery (95% CI 0.22–1.03). Women who planned to save money were 2.48 times more likely to give birth with the assistance of a skilled provider than women who did not plan to save money (95% CI 0.99–6.21) (Table 2).

**Table 2.** Unadjusted and adjusted odds ratios of factors associated with use of skilled provider at birth; Koupéla, Burkina Faso, 2004

Characteristics	Unadjusted odds ratio	95% CI	p value	Adjusted odds ratio	95% CI	p value
Education						
Primary or secondary	1.24	0.431–3.577	0.689	1.21	0.345–4.157	0.757
No. of antenatal care visits	1.52	1.156–1.99	0.003	1.50	1.125–2.002	0.006
Parity						
2–3	0.50	0.200–1.193	0.116	0.44	0.165–1.150	0.094
4–5	0.42	0.159–1.121	0.083	0.40	0.136–1.167	0.093
6 or more	0.51	0.203–1.302	0.161	0.46	0.165–1.260	0.130
Far from health centre	0.44	0.237–0.797	0.007	0.39	0.202–0.756	0.005
Knowledge of at least five danger signs	1.10	0.602–2.017	0.752	1.08	0.552–2.101	0.828
Plan for birth assistant	0.86	0.474–1.558	0.617	0.47	0.217–1.034	0.061
Plan for transport for emergency	0.75	0.959–3.195	0.068	1.71	0.781–3.764	0.179
Plan for saving money for emergency	2.38	1.068–5.300	0.034	2.48	0.988–6.207	0.053

CI=Confidence interval

## DISCUSSION

The findings of the study highlight the relationship between the elements of birth-preparedness and complication readiness and the use of a skilled provider at delivery. Controlling for average distance to health facility, number of antenatal care visits, education of women, and parity, planning to save money was associated with giving birth with the assistance of a skilled provider, although the association was borderline significant (p=0.05). Knowledge of danger signs, planning for a skilled provider at delivery, and planning for transportation in the case of emergency were not significantly associated with giving birth with the assistance of a skilled provider. Few other studies examined the relationship between birth-preparedness and complication readiness and use of skilled providers at delivery.

In this geographical area, women were confident in their ability to locate transport, if needed, and advance planning for transport was, therefore, low. This may also be influenced by the fact that most (88%) women in the sample had access to transportation (bicycle, moped, or car) at home. Programmes should conduct formative research prior to conducting programme interventions to determine if planning for transportation is an appropriate programme message.

There are strengths of and limitations to the study. One criticism of safe motherhood interventions is the failure to adequately measure exposure to programme interventions. In this study, 68.9% of the women were exposed to the MNH Program with the majority exposed through antenatal care. Exposure to the Program was not significantly associated with birthing with the assistance of a skilled provider and was dropped from multivariate analyses.

There are several limitations to this study. First, the study sample was not calculated with adequate power to look at the relationship between birth-preparedness and giving birth with the assistance of a skilled provider, with a sample of 180 women. The qualitative findings support the quantitative data, thus indicating that more research is needed to better understand how birth-preparedness and complication readiness contribute to use of skilled care at delivery. In addition, the study design did not include a control group which limits the ability to make causal statements (13). In future studies, panel designs with a control group may be more effective at more clearly understanding how birth-preparedness and complication readiness influence using a skilled provider at birth.

Finally, there is a debate about the validity and reliability of asking women who have recently given birth to recall a behaviour that occurred prior to childbirth (7). In this study, women were asked about their birth-planning behaviours after delivery. Women were interviewed at 4.5 months postpartum on average, thus reducing recall bias. However, the delivery experience, including obstetric complications that necessitated using services, and the quality of services may have influenced women's recall of birth-preparedness and complication readiness behaviours. In addition, there were significant differences in how women reported birth-preparedness and complication readiness behaviours, depending on phase of the birthing process. Pregnant women were more likely to report planning to give birth with the assistance of a skilled provider than postpartum women, while postpartum women were more likely to report saving money compared to pregnant women. Stanton suggests that validity and reliability could be tested by asking recently-delivered women about birth-preparedness and complication readiness at baseline prior to implementation of programme activities and comparing these responses with birth-preparedness and complication readiness plans at follow-up (7). Unfortunately, questions on birth-preparedness and complication readiness behaviours were not asked of recently-delivered women in the baseline survey. There is a need to further research the validity of asking recently-delivered women about behaviours prior to delivery. Longitudinal or panel samples may be more useful, as pregnant women can be asked about birth-preparedness and complication readiness during pregnancy and followed up after delivery to ascertain if those plans were realized.

Planning to save money for childbirth was associated with using a skilled provider at delivery controlling for other factors, although the association was borderline significant. Currently, safe motherhood programmes are scaling up birth-preparedness and complication readiness interventions to address the first two of the three delays, although there are few studies that have looked at the effects of birth-preparedness and complication readiness on the use of skilled providers at delivery. Although there are several limitations to this study, the results highlight the relationship between birth-preparedness and complication readiness and increasing the use of skilled providers at delivery, underlining the particular importance of saving money for childbirth. There is a need for additional research to further explore these relationships.

## ACKNOWLEDGEMENTS

The authors acknowledge the United States Agency for International Development (USAID) which provided the funding for this survey. The Maternal and Neonatal Health Program was made possible through support provided by the Maternal and Child Health Division, Office of Health, Infectious Diseases, and Nutrition, Bureau for Global Health, under the terms of Award No. HRN-00-98-00043-00. The opinions expressed herein are those of the authors and do not necessarily reflect the views of USAID, the Maternal and Neonatal Health Program, or the Institut Supérieur des Sciences de la Population (ISSP). The authors also acknowledge Mr. Abdoulaye Maiga from ISSP who worked on preliminary data analysis in Burkina Faso.
